# Evolution of the sensitized Er^3+^ emission by silicon nanoclusters and luminescence centers in silicon-rich silica

**DOI:** 10.1186/1556-276X-9-456

**Published:** 2014-09-02

**Authors:** Lingbo Xu, Dongsheng Li, Lu Jin, Luelue Xiang, Feng Wang, Deren Yang, Duanlin Que

**Affiliations:** 1 State Key Laboratory of Silicon Materials and Department of Materials Science and Engineering, Zhejiang University, Hangzhou, 310027, People’s Republic of China

**Keywords:** Luminescence centers, Silicon nanoclusters, Erbium, Energy transfer, Silicon-rich oxide, Photoluminescence

## Abstract

The structural and optical properties of erbium-doped silicon-rich silica samples containing different Si concentrations are studied. Intense photoluminescence (PL) from luminescence centers (LCs) and silicon nanoclusters (Si NCs), which evolves with annealing temperatures, is obtained. By modulating the silicon concentrations in samples, the main sensitizers of Er^3+^ ions can be tuned from Si NCs to LCs. Optimum Er^3+^ PL, with an enhancement of more than two, is obtained in the samples with a medium Si concentration, where the sensitization from Si NCs and LCs coexists.

## Background

There has been a growing interest in developing Si-compatible light sources for photonic applications [1]. A silicon-rich oxide (SRO) matrix embedded with silicon nanoclusters (Si NCs) provides a promising approach, since the quantum confinement effect of Si NCs can overcome the inability of Si bulk with indirect bandgap and thus significantly promote the luminescence efficiency. By utilizing Si NCs, optical gain [2-5] and room temperature electroluminescence [6-10] have been successively demonstrated in the last decades. Moreover, SRO acts as an efficient host matrix for rare-earth ions such as Nd^3+^[11], Tb^3+^[12], and Er^3+^[13]. Among these ions, the Er^3+^ ion has attracted much research interest [14-29], as the 1.54-μm luminescence of Er^3+^ lies in the third telecom window. The Er^3+^ ions in SRO, when located in close proximity to Si NCs, would benefit from energy transfer from Si NCs and subsequently be excited non-resonantly [13,14]. The effective excitation cross section of Si NC-sensitized Er could be several orders of magnitude larger than that of resonantly pumped Er [14], which results from the large and spectrally broad absorption cross section of Si NCs in the entire visible range. Besides, luminescence centers (LCs) in SRO have also been demonstrated to be efficient sensitizers for Er^3+^ ions [18,19]. Thus, the luminescence of Er^3+^ ions can be significantly improved via sensitization of Si NCs or LCs in SRO.

In spite of the advantageous sensitization effect, achieving optical gain from Er^3+^ in SRO has been proved to be a difficult task. This is mainly due to the low fraction of optically active Er^3+^ ions coupled to Si NCs [20,21]. It has been demonstrated that the effective interaction length between Er^3+^ and Si NCs would not exceed 0.5 nm, which means that only the Er^3+^ ions in close proximity to Si NCs could be sensitized [21]. Thus, the relatively low density of Si NCs is believed to be a limitation on achieving gain in such a system. Compared with Si NCs, LCs with high density could be obtained in SRO due to their atomic size scale, and sensitization via LCs is believed to improve the Er^3+^ luminescence. Moreover, some authors attributed the dominant Er sensitization mechanism in Er-doped SRO (SROEr) to LC-mediated excitation [18]. However, this conclusion is under debate, and the sensitization of Si NCs on Er^3+^ ions cannot be totally excluded. In fact, it is necessary to take advantage of all the sensitizers and facilitate their coupling with Er^3+^ ions for the optimization of Er^3+^ luminescence.

In this work, Er-doped SRO films with different Si concentrations were prepared by electron beam evaporation (EBE), and the structural and optical properties were studied. We try to clarify the effect of energy transfer from Si NCs or LCs to the Er^3+^ ions and subsequently optimize the Er^3+^ luminescence.

## Methods

Er-doped SRO films with thicknesses of approximately 400 nm were prepared on *p*-type <100 > silicon substrates by EBE. Three different targets were used: a SiO and Er_2_O_3_ mixed target with Er atomic concentration of approximately 20 at.% was labelled ‘target I’, and target I baked at 80°C in air for 24 and 48 h was labelled ‘target II’ and ‘target III’. Consequently, samples prepared from targets I, II, and III are labelled samples I, II, and III, respectively. The details of the deposition parameters can be found in [19]. Rutherford backscattering spectra were collected using 2.02 MeV ^4^He ion beams at a scattering angle of 165° to determine the atomic compositions of the as-deposited (A.D.) films. The Er concentration was approximately 3 × 10^19^ at./cm^3^ in all the samples, and the Si atomic concentration was 38.5, 37.0, and 35.7 at.% in samples I, II, and III, respectively. After deposition, the samples were placed in a quartz furnace and annealed in flowing N_2_ at temperatures in the range of 700 to 1,150°C for 30 min to form the different sensitizers (LCs and/or Si NCs). The structural characteristics of the annealed samples were studied by transmission electron microscopy (TEM, Tecnai F20G2, FEI Company, Hillsboro, USA). Continuous wavelength photoluminescence (PL) measurements were carried out at room temperature using a 325-nm line of a He-Cd laser as the excitation light source. PL spectra were recorded using a charge-coupled device (PIXIS: 100BR, Princeton Instruments, Trenton, USA) in the visible range and a photomultiplier tube (PMT, Hamamatsu R5509, Iwata City, Japan) in the infrared range. The PL decay traces of the matrices were excited by a 405-nm picosecond laser diode and measured by a multichannel photon counting system (Edinburg Instruments Ltd., Livingston, UK). The Fourier transform infrared (FTIR) investigations were carried out under the transmission mode and vacuum condition using a Bruker IFS 66 V/S FTIR spectroscope (Bruker BioSpin AG Ltd., Beijing, China).

## Results and discussion

**Figure 1:**
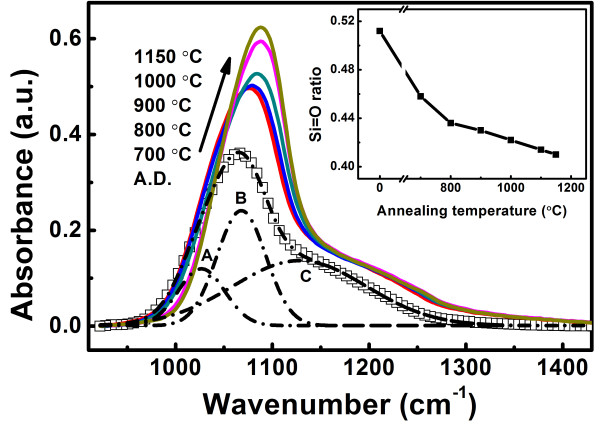
**FTIR spectra and the percentage of Si = O symmetric stretching mode for the SROEr-I films.** FTIR spectra of the SROEr-I films annealed at different temperatures. The spectrum of the as-deposited sample is denoted by empty squares, and that of the annealed samples are denoted by the colored lines. A typical fitting of the FTIR spectra is provided for the A.D. sample (the fitting data are denoted by dash-dot line). The sub-peaks A, B, and C represent the components from Si-O-Si bulk, Si-O-Si surface, and Si = O symmetric stretching modes, respectively. The inset shows the ratio of Si = O symmetric stretching mode for the SROEr-I films with different annealing temperatures.

The structural evolution of sample I with the annealing process was investigated by measuring the FTIR spectra, as shown in Figure 1. The main peak in the range from 1,065 to 1,088 cm^−1^ is ascribed to the Si-O-Si stretching mode [30]. With the increase of annealing temperatures, the intensity of the main peak gradually increases and its peak position shifts to higher wavenumbers, which indicates the phase separation in the SROEr films [31]. In addition, each spectrum can be resolved into three bands with a Gaussian fitting procedure, which are ascribed to the Si-O-Si bulk stretching mode (sub-peak A), the Si-O-Si surface stretching mode (sub-peak B), and the Si = O symmetric stretching mode (sub-peak C) [32]. The ratio of the Si = O symmetric stretching mode in the SROEr films is shown in the inset of Figure 1, and it decreases with the increase of the annealing temperatures, due to the decomposition of the Si = O bonds by Er ions during thermal treatments [19].

**Figure 2:**
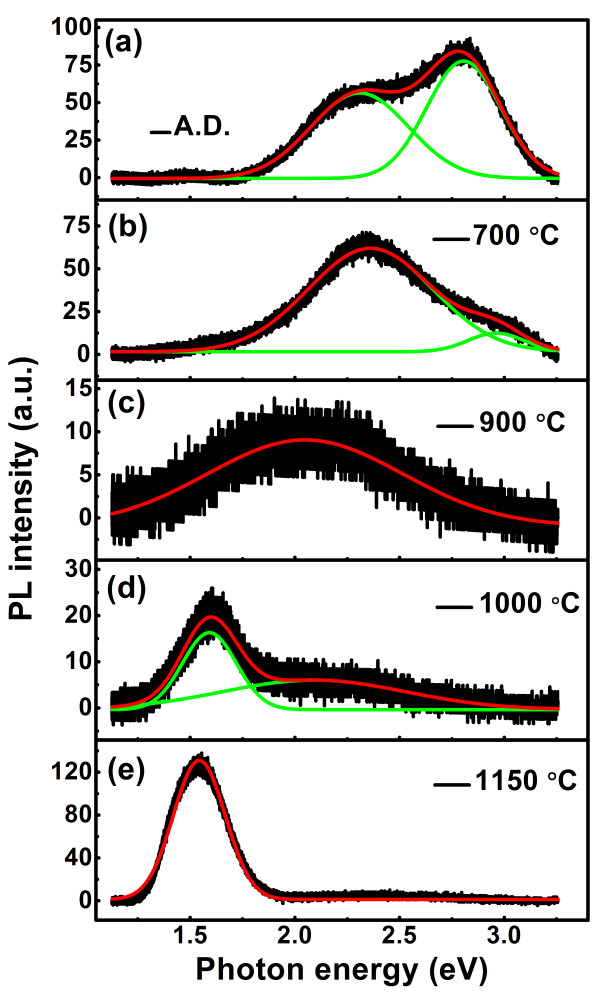
**Matrix-related PL spectra of the SROEr-I films annealed at different temperatures.** PL spectra of the SROEr-I matrix annealed at different temperatures: **(a)** as-deposited, **(b)** 700°C, **(c)** 900°C, **(d)** 1,000°C, and **(e)** 1,150°C. The experimental data are denoted by black lines, and the fitting data of the general and the divided peaks are denoted by the red and green lines, respectively. All the films were excited by the 325-nm line of the He-Cd laser.

Figure 2 shows the matrix-related PL spectra measured from sample I with different thermal treatment processes. Gaussian fittings were applied to these spectra to identify the origin of PL. In the PL spectrum from the as-deposited specimen, two PL bands can be resolved. The PL band with a peak at 2.8 eV originates from weak oxygen bonds (WOBs) [33], and the one with a peak at 2.3 eV originates from Si = O states [19,34]. This is consistent with the results from sample III, as we have studied in [19]. However, the evolution of the PL spectra from sample I with different thermal treatments differs from that of sample III. After annealed at 700°C in N_2_ for 30 min, the PL from WOBs in sample I diminishes while the intensity of PL from Si = O states remains almost constant. Indeed, PL from Si = O states is also unstable during thermal treatments and would disappear after annealed at 900°C, where a weak PL band from non-bridging oxygen hole centers (NBOHCs) arises [35]. When the annealing temperature further increases to 1,000°C, a new PL band peaked at approximately 1.6 eV arises. Stretched exponential fittings of the decay traces have been applied, in the form of *I*(*t*) = *I*_0_exp[-(*t/τ*)^*β*^], where *τ* is the PL decay time and 0 ≤ *β* ≤ 1 is the dispersion exponent. The lifetime of the PL peak at 1.6 eV is about 5 μs, much longer than that of the PL from LCs. Hence, this PL band is considered to originate from Si NCs. Finally, the PL from LCs would disappear, and there is only a PL band from Si NCs in the 1,150°C annealed specimen.

**Figure 3:**
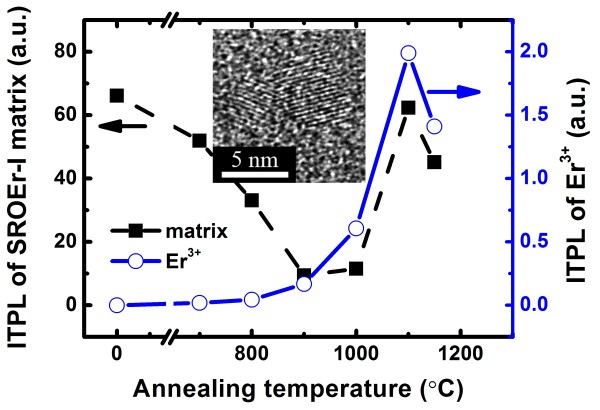
**ITPL of the SROEr-I matrix and Er**^**3+ **^**ions, and HRTEM image of the SROEr-I films.** The evolution of SROEr-I matrix-related ITPL (left-hand scale) and that of Er-related ITPL (right-hand scale) with annealing temperatures. All the films were excited by the 325-nm line of the He-Cd laser. The inset shows the HRTEM image of coalescent Si NCs in the 1,150°C-annealed SROEr-I film.

**Figure 4:**
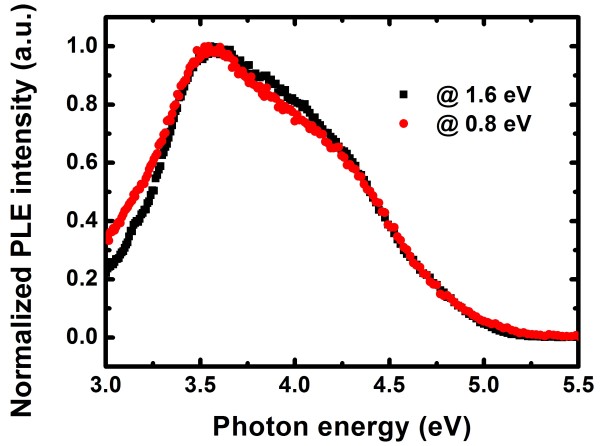
**Normalized PL excitation spectra for the 1,100°C-annealed SROEr-I film.** Normalized PL excitation spectra of Si NCs (measured at 1.6 eV) and Er^3+^ ions (measured at 0.8 eV) for the 1,100°C-annealed SROEr-I film.

Figure 3 shows the integrated PL intensity (ITPL) of SROEr-I matrix and that of Er^3+^ ions (right-hand scale) with different annealing temperatures. In the specimens annealed below 800°C, the PL from Er^3+^ is nearly indistinguishable, though strong PL from LCs (WOBs and Si = O states) can be observed. This suggests that most of the Er^3+^ ions in the low-temperature annealed specimens may be optically inactive and thus could not be sensitized by LCs (WOBs and Si = O states); in other words, high-temperature annealing is required to activate Er^3+^ ions. PL from Er^3+^ ions could only be clearly observed in the specimens annealed above 900°C, and its intensity increases by a factor of 12 with the annealing temperatures until 1,100°C, followed with a sudden drop at 1,150°C. Meantime, the emission decay time for Er^3+^ increases continuously by a factor of 4, from 68 to 287 μs, with the annealing temperature until 1,100°C and it finally reaches 415 μs at 1,150°C. The evolution of the Er^3+^ PL intensity could partially but not solely be explained by the increase of PL decay times. In fact, the evolution of ITPL from Er^3+^ ions with annealing temperatures shows a similar trend to that from the SROEr matrix in the range of 900 to 1,150°C, and the overlap between the two spectra indicates the energy transfer from the matrix to the Er^3+^ ions. By carefully studying the matrix-related PL, energy transfer from NBOHCs to Er^3+^ ions is excluded due to their gradual decrease and eventual absence during high-temperature annealing (900 to 1,150°C), which is in contrast to the evolution of ITPL from Er^3+^ ions. Therefore, Si NCs are believed to be the main sensitizers for Er^3+^ ions in the high-temperature annealed SROEr-I films. Indeed, the PL excitation (PLE) spectrum of Er^3+^ in the 1,100°C-annealed SROEr-I film shows similar wavelength dependence to that of Si NCs (as shown in Figure 4), confirming our hypothesis. The degradation of ITPL from Er^3+^ ions and matrix in the 1,150°C-annealed specimen is ascribed to the growth and coalescence of Si NCs, as shown in the inset of Figure 3. Coalescent microstructures of Si NCs, which would reduce the density of sensitizers and limit the non-phonon recombination probability in Si NCs, have been demonstrated to be detrimental for the luminescence of Si NCs and Er^3+^ ions [22,36].

**Figure 5:**
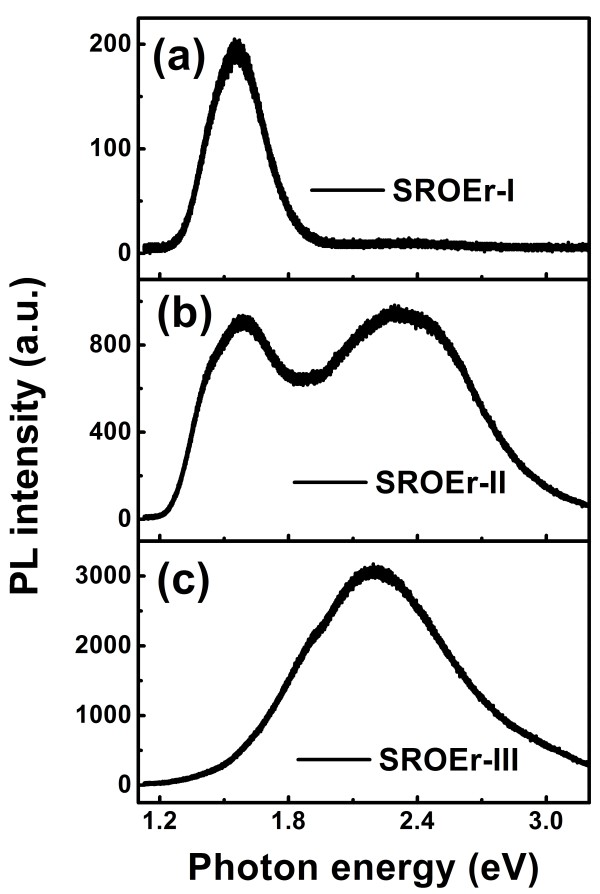
**Matrix-related PL spectra of the 1,100°C-annealed SROEr films with different Si concentrations.** Matrix-related PL spectra of samples with different Si concentrations: **(a)** SROEr-I, 38.5 at.%, **(b)** SROEr-II, 37.0 at.%, and **(c)** SROEr-III, 35.7 at.%. All the films were excited by the 325-nm line of the He-Cd laser.

Figure 5 shows the matrix-related PL spectra measured from the 1,100°C-annealed SROEr films with different Si concentrations. Two PL bands can be resolved from the PL spectra: one peaked at approximately 1.6 eV, which originates from Si NCs; and the other peaked at approximately 2.3 eV, which originates from Si = O states, as described in [19]. In sample I with the highest Si concentration, PL from Si NCs dominates, while there is only PL from LCs in sample III with the lowest Si concentration. In sample II with a medium Si concentration, PL from Si NCs and LCs coexists and has comparable intensities. Remarkably, matrix-related PL spectra from SROEr films could be modulated by changing the Si concentrations, and this phenomenon deserves a special discussion. Si = O states, embedded in amorphous SROEr network or at the surface of Si NCs, act as efficient light emitters, and their photon absorption would be facilitated by the introduction of Si NCs [19]. However, growth and coalescence of Si NCs in samples with high Si concentrations would deplete the network where Si = O states are embedded and consequentially reduce the density of Si = O states [37]. Besides, local stress induced by agglomeration of Si NCs could dynamically destroy Si = O bonds and it is more severe in samples with higher Si concentrations [38]. Hence, we consider that the modulation of matrix-related PL originates from the evolution of microstructures with Si concentration in SROEr films.

**Figure 6:**
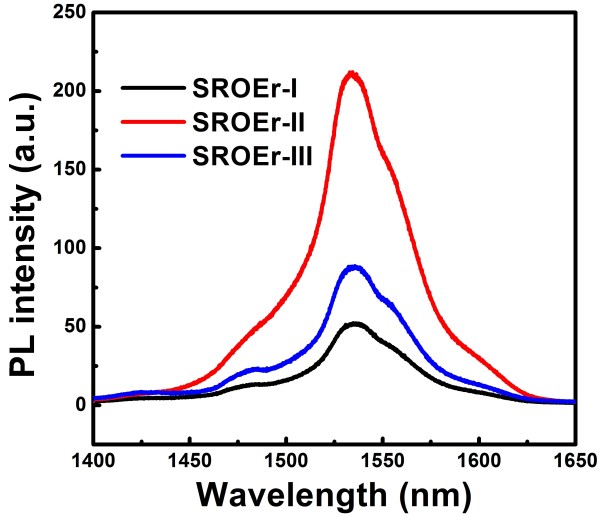
**Er-related PL spectra for the SROEr films with different Si concentrations.** PL spectra of Er^3+^ for the 1,100°C-annealed SROEr films with different Si concentrations. All the films were excited by the 325-nm line of the He-Cd laser.

Meanwhile, sensitization mechanism of Er^3+^ ions in SROEr films evolves with the Si concentrations as a result of the modulation of matrix-related PL. In sample I, Si NCs play the dominant role in sensitizing Er^3+^ ions, and in sample III, LCs would take the place of Si NCs [19]. When it comes to sample II, it is reasonable to expect that both mechanisms would take place and may compete with each other. Due to the atomic size scale, LCs with high density could be obtained and are most often considered as sensitizers more promising than Si NCs. It is hence expected that the stronger PL from LCs is achieved, the more effective sensitization of Er^3+^ ions will be acquired. However, our data show that this is not so straightforward. The PL spectra of Er^3+^ ions from the 1,100°C-annealed SROEr films with different Si concentrations are presented in Figure 6. It is shown that Er^3+^ PL from sample III is stronger than that from sample I, indicating that LCs do be effective sensitizers for Er^3+^ ions. However, in spite of the relatively weak LC-related PL, Er-related PL in sample II is much stronger than that in sample III. This confirms our hypothesis that both mechanisms of sensitization via Si NCs and LCs take place in sample II. Moreover, it suggests that not only the density of sensitizers is a limitation for Er^3+^ luminescence, but also the coupling between sensitizers and Er^3+^ ions is important. According to Förster's theory [39,40], the energy transfer probability depends on the distance between sensitizers and Er^3+^ ions. The coupling between sensitizers and Er^3+^ ions would evolve with the modulation of microstructures for samples with different Si concentrations, and the optimization is expected for sample II with a medium Si concentration.

## Conclusions

In conclusion, we studied the modulation on the sensitization mechanism of Er^3+^ ions as well as the matrix-related luminescence by the composition evolution of SROEr films. Formation of LCs and Si NCs is found to depend on the Si concentrations: growth and coalescence of Si NCs are promoted with the increase of Si concentrations, depleting the network where LCs are embedded and quenching the LC-related PL. Subsequently, the sensitization mechanism of Er^3+^ ions evolves with the modulation of microstructures. Si NCs act as main sensitizers of Er^3+^ ions in samples with high Si concentrations, and LCs take their place in samples with low Si concentrations, while the sensitization mechanisms coexist in samples with medium Si concentrations. An optimum Er^3+^ PL with an enhancement of more than two was obtained for the sample with medium Si concentrations, where the coupling between Er^3+^ ions and sensitizers (Si NCs and LCs) was maximized.

## Abbreviations

A.D.: as-deposited; EBE: electron beam evaporation; FTIR: Fourier transform infrared; ITPL: integrated PL intensity; LCs: luminescence centers; NBOHCs: non-bridging oxygen hole centers; PL: photoluminescence; PLE: photoluminescence excitation; PMT: photomultiplier tube; Si NCs: silicon nanoclusters; SRO: silicon-rich oxide; SROEr: erbium-doped silicon-rich oxide; TEM: transmission electron microscopy; WOBs: weak oxygen bonds.

## Competing interests

The authors declare that they have no competing interests.

## Authors’ contributions

LX participated in the sample preparations and measurements, analyzed the data, and drafted the manuscript; DL conceived the study, participated in the result discussion, and revised the manuscript; LJ carried out the experiments and measurements and participated in the result discussion; LX, FW, DY, and DQ contributed to the analysis of the results in this paper. All authors read and approved the final manuscript.
